# Frontal disconnection surgery for drug‐resistant epilepsy: Outcome in a series of 16 patients

**DOI:** 10.1002/epi4.12424

**Published:** 2020-08-14

**Authors:** Hamda Kamalboor, Hindi Alhindi, Faisal Alotaibi, Ibrahim Althubaiti, Mashael Alkhateeb

**Affiliations:** ^1^ King Faisal Specialist Hospital & Research Center Riyadh Saudi Arabia; ^2^ Rashid Hospital Dubai United Arab Emirates

**Keywords:** frontal, disconnection, epilepsy, surgery, outcomes

## Abstract

**Objective:**

To evaluate the effectiveness of frontal disconnection surgery in seizure control and related consequences in a consecutive patient series.

**Methods:**

We conducted a retrospective analysis of patients who underwent frontal disconnection surgery for drug‐resistant epilepsy (DRE). Baseline epilepsy characteristics, detailed presurgical evaluation including epileptogenic zone (EZ) localization, magnetic resonance imaging (MRI) detection of epileptogenic lesion, and pathological findings were reviewed. Patients were followed postoperatively for seizure outcome at 1 year.

**Results:**

A total of 16 patients were identified (six children and 10 adults). Most patients had a childhood onset of DRE with a median duration of epilepsy of 6.5 years (interquartile range 3.5‐17.5 years) before surgery. In 10 (62.5%) patients, the EZ was localized to the frontal lobe, while in six patients, the EZ involved also adjacent lobes or consisted of multiple foci. In 10 (62.5%) patients, an epileptogenic lesion was detected on presurgical MRI, four of which (40%) had all MRI abnormalities confined to the frontal lobe. Two‐thirds of the patients (11/16; 68.8%) underwent isolated frontal disconnection procedure, while remaining patients had frontal disconnection combined with resection of an adjacent lobe. Of the 12 patients in whom biopsy was taken from the disconnected frontal lobe, six (50%) had pathology‐proven focal cortical dysplasia. We observed surgical‐related complications in three (18.8%) cases, neurological deficits in other three (18.8%) patients, and worsening cognitive abilities in one (6.3%) patient. Overall, eight (50%) patients became completely seizure‐free (ILAE 1) at one‐year follow‐up.

**Significance:**

Frontal disconnection surgery for DRE can result in seizure freedom in certain patients, especially when the EZ is strictly limited to the ipsilateral frontal region, and the MRI shows an epileptogenic lesion that is purely frontal in location. Frontal lobe disconnection procedure is safe and has a limited complication rate. However, further studies with larger patient population will yield more significance.

**Key Points**

- Frontal disconnection surgery for drug‐resistant epilepsy can result in seizure freedom in certain patients.
- Frontal cortical dysplasia is the most common underlying etiology in patients with drug‐resistant epilepsy arising from frontal lobe.
- Frontal lobe disconnection procedure is safe and has a limited complication rate.

## 1 INTRODUCTION

Frontal lobe surgery is the second most common surgical intervention for focal drug‐resistant epilepsy (DRE) after temporal lobectomy.^1^ However, it is traditionally a resection‐type surgery, removing large amounts of brain parenchyma. Histopathologic evaluation showed an underlying focal cortical dysplasia (FCD) in more than 50% of cases.^2^, ^3^

The outcome of resective type of frontal lobe surgery varied greatly with regard to postoperative seizure freedom that ranged between 25% and 69%.^2^, ^4^, ^5^, ^6^, ^7^, ^8^ Since FCD is the most common underlying etiology in DRE arising from frontal lobe and magnetic resonance imaging (MRI) studies in cases of FCD can be normal or associated with subtle abnormalities, this may explain some of the seizure recurrences and variable seizure outcomes across the studies. Thus, surgical planning ultimately relies on clinical and electroencephalographic (EEG) evaluation rather than structural neuroimaging.

Some patients would require invasive EEG evaluation to determine the boundaries of the epileptogenic zone (EZ) that might be beyond that appreciated on structural imaging in order to improve the accuracy of the planned resection, which in turn can improve the surgical outcome even in MRI‐negative patients.^9^ However, this can result in a large resective procedure leaving behind a large surgical cavity, which was previously thought to be associated with hydrocephalus, hematoma in the residual cavity, and superficial hemosiderosis in cases of other types of resective surgeries with large residual cavity such as hemispherectomy and posterior quadrantectomy.^10^, ^11^ This has led to the development of disconnective techniques such as hemispheric deafferentation or functional hemispheric disconnection, hemispherotomy, and posterior quadrant disconnection.^10^, ^12^, ^13^

Current disconnection surgeries for DRE include functional hemispheric disconnection, corpus callosotomy, temporal disconnection, and posterior quadrant disconnection. Posterior quadrant disconnection was reported in few clinical series.^10^, ^14^, ^15^, ^16^, ^17^, ^18^, ^19^, ^20^ We have come across only two surgical series in the literature involving temporal disconnection.^11^, ^21^ On the other hand, frontal disconnection surgery has not been reported in a clinical series before; as per our review, it has only been reported in two case reports, one in an adult patient and another in a pediatric patient.^22^, ^23^ We consider frontal, temporal, and posterior quadrant disconnection surgeries as subtypes of “lobar disconnection surgery.” Posterior quadrant disconnection further subtypes include parietooccipital, temporoparietooccipital, and temporooccipital disconnection surgeries.

Based on the concept that the surgical interruption of fibers in an epileptic network through disconnection can result in a similar effect to that seen in resective surgery,^10^ which has been applied in prior studies involving disconnection of posterior quadrant and temporal lobe, we applied disconnection of frontal lobe in our series.

In this study, we describe our experience with frontal disconnection surgery as an alternative surgical technique to frontal resection and evaluate its effectiveness in seizure control and related consequences.

## 2 METHODS

### 2.1 Study population

In this retrospective study, we have included all consecutive patients with DRE who underwent frontal disconnection surgery following routine presurgical evaluation in our comprehensive epilepsy program at King Faisal Specialist Hospital and Research Center (KFSH&RC), Riyadh, Kingdom of Saudi Arabia, from January 2017 to October 2018 and have completed a one‐year follow‐up. We have retrospectively identified a total of 16 patients. Ethical approval has been obtained from the research center review board committee at KFSH&RC.

All patients had standard presurgical epilepsy evaluation that is routinely done for any patient with DRE in order to determine their candidacy for epilepsy surgery and to decide on the appropriate surgical option. It included a detailed clinical evaluation, formalized neuropsychological testing, prolonged scalp video‐EEG monitoring, high‐resolution brain MRI, and brain positron emission tomography (PET) scan using fluorodeoxyglucose (^18^F‐FDG). All data were presented and evaluated in the Epilepsy Management Conference, a multidisciplinary meeting. The result of the meeting determined the appropriate course of action, either a decision on the type of the surgical intervention or a recommendation to proceed with invasive EEG monitoring using subdural electrodes, sometimes with the addition of depth electrodes.

The surgical intervention that was decided in our 16 patients consisted of either isolated frontal disconnection or frontal disconnection combined with resection of an adjacent lobe.

### 2.2 Baseline epilepsy characteristics

At baseline and prior to surgery, all patients were assessed for age of seizure onset, duration of epilepsy, number of seizure types, seizure frequency, and presence of neurological deficits and other comorbidities. The number of seizure types was classified into single, two, or multiple types. Seizure frequencies were divided into: (a) daily, (b) weekly more than 3 days, (c) weekly 3 days or less, and (d) monthly 3 days or less. Patients with epilepsy may have a wide range of comorbidities that vary in type and severity. This has been emphasized in the latest update of International League Against Epilepsy (ILAE) classification of the epilepsies.^24^ In our study, we have considered mainly cognitive, behavioral, developmental, and psychiatric comorbidities. These were retrieved from the neuropsychology evaluations and categorized as: (a) single cognitive domain involvement, (b) more than one cognitive domain involvement or intellectual disability, (c) developmental disorder, (d) psychiatric or behavioral disorder, and (e) combination of any of the above. We have separated neurological deficits from the other comorbidities, since the latter reflects a more widespread functional deficit than that expected with focal neurological deficits such as limb weakness, dysarthria, or visual field defects.

During epilepsy monitoring unit (EMU) evaluation, seizure semiologies and associated scalp and/or invasive EEGs were reviewed for EZ(s) localization and hemispheric lateralization. In cases of multifocal epilepsy, the most disabling seizure arising from a clearly identified EZ was chosen for surgical intervention.

### 2.3 Neuroimaging

All patients had their presurgical MRI at 1.5 or 3 Tesla performed between 2015 and 2018. Brain MRI studies consisted of the following sequences: sagittal T1‐weighted image, axial and coronal T2‐weighted images, axial and coronal fluid‐attenuated inversion recovery (FLAIR) images, axial gradient echo (GRE) T2*‐susceptible image or susceptibility‐weighted image (SWI), coronal inversion recovery (IR) image, and axial 3‐dimensional (3D) T1‐weighted fast spoiled gradient echo (FSPGR) with brain volume (BRAVO) image. Additional sequences for some cases may have included axial diffusion‐weighted image (DWI), axial apparent‐diffusion coefficient (ADC) image, axial IR image, and coronal and sagittal 3D T1‐weighted FSPGR with BRAVO images. Contrast medium was administered in 3 cases (no. 1, 8, and 11) during presurgical MRI study.

In our study, presurgical MRIs were rated based on documented radiological reports by neuroradiologists for the presence or absence of lesional brain abnormalities and whether the epileptogenic lesion is frontal or extra‐frontal, that is, extending beyond the frontal lobe.

### 2.4 Surgical technique

Under general anesthesia with endotracheal intubation, the patient is placed in a supine position with head tilt to the contralateral side. Neuronavigation system is applied for this procedure in certain cases. A curvilinear skin incision is made covering the entire frontal area. Craniotomy is created over the frontotemporal area exposing the perirolandic and opercular areas. Then, dura is opened in a U‐shaped or cruciate manner. In case of a previous skin incision and craniotomy performed for implantation of subdural EEG electrodes, the same approach is utilized. At this point, we identified the central and precentral sulci in order to mark the posterior edge of the planned frontal disconnection, which would be anterior to the precentral gyrus and sulcus (anterior to the motor cortex). However, in cases of previously implanted subdural grids and strips, these were used to identify the EZ by localizing the involved contacts as per the invasive EEG study in order to guide the posterior border of the planned frontal disconnection. Then, the subdural electrodes were removed. In certain patients where the posterior border of the EZ was estimated to be in close proximity to the primary motor cortex, we performed neurophysiological intraoperative brain mapping of the primary sensorimotor cortices to confirm central sulcus location in two steps. First, a subdural strip of 8‐contact platinum electrode (AD TECH) is positioned over the cerebral cortex and a contralateral median somatosensory evoked potentials (SSEPs) phase reversal technique is used to localize the central sulcus. During the second part, the surgeon applies direct cortical and subcortical stimulation (monopolar probe, five‐pulse train, 50 Hz, 200 msec pulse duration, 5‐25 mA intensity) to trigger motor evoked potentials (MEPs) in the monitored muscles in order to confirm the location of the motor cortex.

**FIGURE 1:**
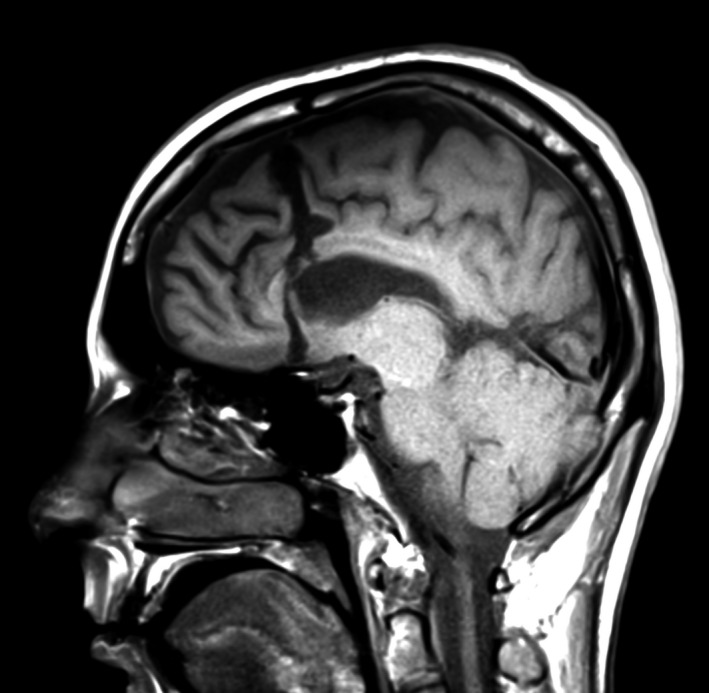
Postsurgical MRI including sagittal T1‐weighted image that demonstrates the frontal disconnection line through the anterior corpus callosum

The disconnection plane is marked anterior to the precentral sulcus. The motor cortex was covered and protected by a surgical patty. Using the surgical microscope, the disconnection is started with a gyrotomy of the cortex at the superior and middle frontal gyri, anterior to the precentral sulcus, followed by subpial dissection using cavitron ultrasonic surgical aspirator (CUSA) device in a medial direction through the white matter, mesial frontal gyri and cingulate gyrus until the falx and corpus callosum are exposed. Anterior corpus callosotomy is performed involving the rostrum of the corpus callosum while preserving both the callosomarginal and pericallosal arteries and using the A2 segment of anterior cerebral artery as a landmark. Then, the direction of the disconnection descended laterally to the frontal horn or body of the lateral ventricle and down to the base of the frontal lobe dissecting the rectus gyrus and exposing the olfactory nerve, which is the inferior landmark that would be left intact, thus performing the medial part of the frontobasal disconnection line. Going back to the dorsolateral frontal surface of the disconnection plane, dissection is carried out in the lateral direction to the inferior frontal gyrus (at the suprasylvian frontal operculum), just anterior to the facial motor representation areas in a nondominant hemisphere, and then down through the white matter disconnecting the frontal lobe from the insula and caudate head and continuing inferiorly until reaching the orbitofrontal area and exposing the edge of the sphenoid wing. The previously done frontobasal disconnection line is reached from the lateral side, thus completing the disconnection (Figure 1). In three patients with dominant frontal lobe for language, the inferior frontal gyrus is preserved at the dorsolateral disconnection line and the dissection is done anterior to the orbitalis gyrus.

The dura is closed in a continuous watertight manner, and the bone flap is fixed back using microplates and screws. Then, the temporalis muscle is closed in an interrupted inverted manner, the subcutaneous tissues and galea using stitches, and the skin using a stapler.

In certain cases, the frontal disconnection procedure was combined with resection of an adjacent lobe, such as the temporal or parietal lobes, during the same surgery (Figure S1). This was based on the extent of the EZ identified during the invasive EEG study. No intracranial drainage tubes were placed for this procedure.

### 2.5 Histopathology

The surgical tissue specimens were examined and dissected by a neuropathologist. In all cases, all tissues were processed for microscopic evaluation. Routine eosin and hematoxylin stain (H&E) was used and supplemented by immunohistochemical stains as needed including neuronal nuclear antigen (Neu‐N), neurofilament protein (NFP), and glial fibrillary acidic protein (GFAP). Neuropathologist (HA) reviewed and categorized the pathological diagnoses of all cases according to current recommendations.^25^, ^26^, ^27^

### 2.6 Postoperative follow‐up

At immediate postoperative period, patients were evaluated for surgical complications. During the postoperative follow‐up period, patients were seen approximately every 3‐6 months for clinical evaluation including assessment of seizure outcome.

For this retrospective study, we evaluated seizure outcome at one year postoperatively using the ILAE classification^28^ and assessed for the occurrence of complications at the perioperative period including surgical‐related complications and postoperative neurological deficits. Postoperative formal neuropsychological evaluation was not performed in the majority of these patients during follow‐up, and we depended on patient or caregiver reporting during clinic follow‐up visits; in cases of reported worsening, an earlier scheduled neuropsychology postoperative evaluation (ie, within 9‐12 months) would be prompted.

Apparently, a good predictor of long‐term seizure freedom state following epilepsy surgery is seizure freedom at 6 months to 2 years postoperatively.^6^ We have chosen one year rather than 6 months, because most (75%–80%) of the seizure recurrences occur during the first 6 months postoperatively.^4^, ^5^

### 2.7 Data collection

We retrieved and collected data on the variables we intended to investigate from patient charts including reports from clinic visits and EMU admissions, MRI and pathology reports, and operative reports.

### 2.8 Statistical testing

We performed statistical testing on IBM SPSS Statistics version 24. Descriptive statistics were obtained for each variable including medians and interquartile ranges for continuous variables and frequencies and percentages for categorical variables. The data were analyzed using Wilcoxon rank‐sum, chi‐square, and Fisher's exact tests to compare patients who achieved seizure freedom at 1 postoperative year to those who did not. Similarly, analysis was performed to compare patients who developed surgical‐related complications to those who did not. Results were considered statistically significant at the 5% level.

## 3 RESULTS

### 3.1 Patient demographics

A total of 16 patients were retrospectively identified including six children (≤18 years of age) and 10 adults. Eleven of the 16 (68.8%) patients were male. The median age at surgery was 21.5 years (interquartile range 14.5‐29.5 years). One patient (no. 8) had previous epilepsy surgery consisting of right selective amygdalohippocampectomy (Table 1).

**TABLE 1:** Patients’ clinical and surgical characteristics

No	Age at epilepsy onset (y)	Age at surgery (y)	Seizure types	Baseline seizure frequency	EZ	Neurodisability/ Comorbidities	Lesional MRI	PET	Surgery type, side	Pathologic findings	Periop complication	Postop neurodisability	Second surgery	ILAE at 12m
1	3	38	Single seizure type	Weekly 3 days or less	R, frontal^a^ (inferior dorsolateral maximum with field extending to superior margin of structural lesion)	None/ Single cognitive domain	Yes (frontal)	R frontal lobe hypometabolism more within superior frontal area	Disconnection, R	MCD (FCD IIb)	None	No change from baseline	No	1a
2	0.5	2	Single seizure type	Daily	R, frontal (prefrontal with frontopolar maximum and a broad field over R hemisphere)	None/ Developmental disorder	Yes (frontal)	R inferior frontal area hypometabolism	Disconnection, R	MCD (FCD IIb)	None	New deficit	No	1a
3	0.5	18	Two seizure types	Daily	R, frontoparietal^a^ (perirolandic; mesial frontal, mesial and superior dorsolateral parietal)	None/ More than one cognitive domain or ID	Yes (extra‐frontal)	B/L symmetrical medial temporal areas and R postcentral gyrus of parietal lobe hypometabolism	Disconnection, R	MCD (FCD IIb)	Intraoperative bleeding requiring blood transfusion	No change from baseline	No	4
4	6	21	Two seizure types	Weekly 3 days or less	B/L, multiple foci^a^ (R frontopolar as most active, L temporal)	None/ Combination of any	No	R more than L medial temporal area hypometabolism	Disconnection, R	No specific changes found	None	New deficit	No	1a
5	29	32	Single seizure type	Monthly 3 days or less	L, frontal^a^ (frontopolar and orbitofrontal with rapid spread to L temporal)	None/ More than one cognitive domain or ID	Yes (extra‐frontal)	L mesiopolar temporal area hypometabolism	Disconnection, L	Glial scar	None	No change from baseline	Yes, VNS	5
6	9	13	Single seizure type	Daily	R, frontal (broad dorsolateral‐mesial field)	None/ More than one cognitive domain or ID	No	Normal	Disconnection, R	MCD (FCD IIa)	None	Worsened baseline cognitive deficits	Yes, resection	5
7	10	16	Single seizure type	Weekly 3 days or less	R, frontal^a^ (middle and posterior dorsolateral possibly involving primary motor cortex with rapid mesial spread)	None/ Combination of any	No	R anteromedial frontal lobe and B/L (mild) medial temporal area hypometabolism	Disconnection, R	No specific changes found	Intracranial hemorrhage	No change from baseline	Yes, VNS	5
8	6	24	Single seizure type	Daily	R, multiple foci^a^ (anterior dorsolateral frontal, mesial frontal, and inferior parietooccipital)	Visual/ Combination of any	Yes (extra‐frontal)	R cerebral hemisphere hypometabolism especially R occipital parasagittal area	Disconnection, temporal resection and parietooccipital MST, R	Glial scar	Infection	No change from baseline	No	5
9	20	37	Single seizure type	Weekly 3 days or less	L, frontotemporal^a^ (superior temporal gyrus and orbitofrontal synchronously)	None/ Single cognitive domain	Yes (frontal)	Small area around cavernoma in L frontal lobe and B/L (subtle) medial temporal area hypometabolism	Disconnection and temporal resection, L	Frontal cavernoma (radiologically) and no specific changes found in temporal lobe	None	No change from baseline	No	1a
10	11	22	Single seizure type	Weekly 3 days or less	R, frontoparietal^a^ (perirolandic; superior dorsolateral frontal and frontopolar, and mesial parietal)	Motor/ Not documented	Yes (extra‐frontal)	R frontal lobe hypometabolism especially at peri‐insular and superior‐anterior parasagittal area, as well as adjacent R superior parietal area and R mesiopolar temporal area	Disconnection and parietal resection, R	MCD (FCD Ia)	None	No change from baseline	No	4
11	19	26	Single seizure type	Weekly more than 3 days	R, frontal^a^ (mesial and dorsolateral)	None/ More than one cognitive domain or ID	No	Small area in R frontal lobe and B/L (subtle) medial temporal area hypometabolism	Disconnection, R	No tissue submitted	None	No change from baseline	No	1a
12	22	27	Two seizure types	Weekly 3 days or less	R, frontal^a^ (mesial and dorsolateral)	None/ More than one cognitive domain or ID	No	B/L (subtle) medial temporal area hypometabolism	Disconnection, R	No tissue submitted	None	No change from baseline	No	5
13	11	17	Single seizure type	Daily	R, frontal (prefrontal, possibly also including frontopolar and orbitofrontal)	Speech/ More than one cognitive domain or ID	Yes (extra‐frontal)	R frontal, temporal and parietooccipital areas hypometabolism	Disconnection and temporal resection, R	Dual pathology (HS and glial scar)	None	New deficit	No	1a
14	0.7	2.9	Two seizure types	Daily	R, frontal^a^ (diffuse mesial and dorsolateral)	None/ Combination of any	Yes (frontal)	(Ictal PET imaging) hypermetabolism at R anterior mid frontal lobe and global surrounding hypometabolism of R frontal lobe and to a lesser degree at L frontal parasagittal region	Disconnection, R	No specific changes found	None	No change from baseline	No	1
15	9	57	Single seizure type	Weekly more than 3 days	R, frontal^a^ (mesial with rapid spread to dorsolateral and frontobasal)	None/ Combination of any	No	Normal	Disconnection, R	No specific changes found	None	No change from baseline	No	4
16	11	13	Single seizure type	Daily	L, frontotemporal^a^ (orbitofrontal and anterior dorsolateral frontal, and posterior middle and inferior temporal gyri)	None/ Combination of any	Yes (extra‐frontal)	L especially lateral more than R medial temporal area hypometabolism	Disconnection and temporal resection, L	MCD (FCD IIa)	None	No change from baseline	No	1

Abbreviations: B/L, bilateral; EZ, epileptogenic zone; FCD, focal cortical dysplasia; ID, intellectual disability; L, left; MCD, malformations of cortical development; MRI, magnetic resonance imaging; MST, multiple subpial transections; R, right; VNS, vagal nerve stimulator.

^a^ Underwent invasive EEG with implanted subdural electrodes.

### 3.2 Baseline epilepsy characteristics

Most patients had childhood onset of DRE with median age at epilepsy onset of 9.5 years (interquartile range 4.5‐15 years). The median duration of epilepsy before surgery was 6.5 years (interquartile range 3.5‐17.5 years). Most patients (12/16; 75%) had single seizure type. In seven (43.8%) patients, the baseline seizure frequency was daily, and in six (37.5%) patients, it was weekly for 3 days or less.

In 10 (62.5%) patients, the EZ was localized to frontal lobe, while in remaining patients, the EZ additionally involved adjacent lobes in four patients (25%; two parietal and two temporal) and involved multiple foci in two patients (12.5%; one as bi‐hemispheric and one as uni‐hemispheric). The EZ in most cases (12/16; 75%) was lateralized to the right hemisphere.

Three (18.75%) patients had baseline neurological deficits including one with motor deficit, one with speech abnormality, and one with visual field deficit, which resulted from previous temporal lobe epilepsy surgery. On review of neuropsychological evaluation prior to surgery that was available in 15 cases, baseline comorbidities included involvement of a single cognitive domain in two (13.3%) patients, involvement of more than one cognitive domain in six (40%) patients, developmental disorder in one (6.7%) patient, and combination of neuropsychiatric comorbidities in six (40%) patients.

### 3.3 Neuroimaging findings

Six (37.5%) patients were MRI‐negative, while in 10 (62.5%) patients, an epileptogenic lesion was detected on presurgical MRI. In four of 10 patients (40%), all MRI abnormalities were confined to the frontal lobe, while remaining six patients (60%) had neuroimaging evidence of “extra‐frontal” abnormalities suggesting a more extensive hemispheric pathology beyond the frontal lobe. This included ipsilateral insular cortical abnormalities in two patients, ipsilateral temporal abnormalities in three patients, and ipsilateral temporooccipital abnormalities in one patient.

### 3.4 Surgical planning and intervention

Most of the patients (13/16; 81.3%) underwent invasive EEG evaluation with chronically implanted subdural electrodes, two of which underwent neurophysiological intraoperative sensorimotor brain mapping (no. 7 and 8).

Two‐thirds of the patients (11/16; 68.8%) underwent isolated frontal disconnection procedure (Figure 1). In the remaining five (31.3%) patients, frontal disconnection was combined with a resection of an adjacent lobe including temporal resection in four patients and parietal resection in one patient. In one of the patients (no. 8) with multiple epileptogenic foci who underwent frontal disconnection combined with temporal tissue resection, an additional procedure involving multiple subpial transections was performed at the parietooccipital cortex that was involved in the EZ.

### 3.5 Histopathologic findings

Surgical specimens were available for 14 patients, of which 12 were obtained from disconnected frontal lobe and two were obtained only from additionally resected temporal tissue (no. 9 and 13). Of the 12 patients in whom biopsy was taken from the disconnected frontal lobe, FCD was found in six (50%) patients (one type Ia, two type IIa, and three type IIb). In the remaining six patients, glial scar was found in two patients and no specific pathological changes in remaining four patients. Frontal lobe pathology in the two patients whom the biopsy was only obtained from the additionally resected temporal tissue was based on radiological finding of frontal cavernoma in one patient and glial scar in the other that had a similar radiological appearance to the resected temporal lobe with pathology‐proven glial scar, in addition to hippocampal sclerosis.

One of the patients (no. 6) had no specific pathological changes on first surgical specimen from frontal disconnection procedure; however, following a second surgery with extended frontal lobe resection following mapping of EZ by chronic subdural EEG recording, a second surgical specimen revealed FCD type IIa.

Of the six MRI‐negative patients, four specimens were available for histopathologic examination that revealed FCD type IIa in one patient (25%), which was demonstrated in the surgical specimen from the second surgery as mentioned above. Nonspecific pathological findings were seen in the remaining three (75%) patients.

### 3.6 Surgical complications

We observed serious surgical complications in three cases (18.8%). One patient (no. 8), who had a previous epilepsy surgery for right selective amygdalohippocampectomy, developed a right subgaleal and epidural abscess presenting with asymptomatic swelling at the surgical site that worsened due to antibiotic noncompliance by the patient requiring redo craniotomy for evacuation and debridement. Eventually, he underwent a second surgery involving right‐sided wound debridement and removal of the infected skull bone flap. During the long period of treating his infection complication, the patient reported that his seizures were occurring at a similar frequency to preoperative baseline. However, at 22‐month follow‐up, the patient presented in good overall condition with healed wound and reached a state of around 50% seizure reduction. Another patient (no. 7) had a frontal epidural hematoma presenting with new semiology of seizures necessitating hematoma evacuation. The third patient (no. 3) had significant intraoperative bleeding requiring blood transfusion; however, he did not develop any apparently related sequela.

We observed neurological deficits in three patients (18.8%) that presented mainly as contralateral mild limb weakness due to involvement of supplementary or primary motor area. One patient (6.25%; no. 6) with baseline comorbidity involving impairment in more than one cognitive domain had experienced worsening cognitive abilities following right‐sided frontal disconnection without any change in postoperative seizure frequency. Formal postoperative neuropsychology evaluation in this patient done prior to reoperation revealed mild worsening in auditory‐verbal learning and memory, as well as slight worsening in visuospatial skills. None of the patients reported perceived language deficits postoperatively. However, no formal postoperative neuropsychological evaluation was available for the three patients who underwent frontal disconnection on the dominant side that might reveal unnoticed language abnormalities. No patient experienced hydrocephalus postoperatively.

There were no statistically significant differences in the baseline clinical, neuroimaging, surgical, and histopathologic variables in those who developed surgical‐related complications compared to those who did not.

### 3.7 Seizure outcomes

**FIGURE 2:**
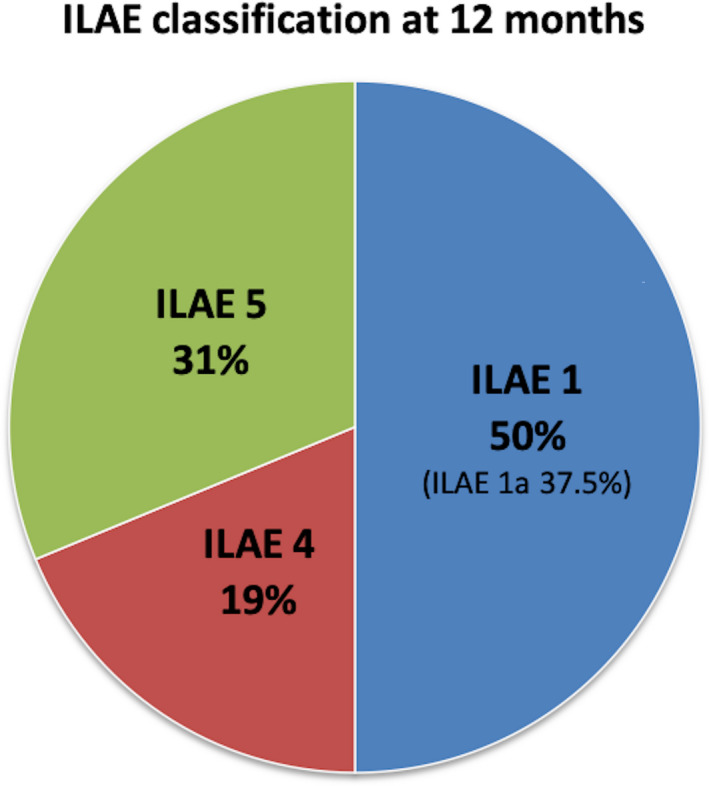
Seizure outcome at 12 months using International League Against Epilepsy (ILAE) classification

Overall, eight (50%) cases became completely seizure‐free (ILAE 1) at one‐year follow‐up with six (37.5%) patients being completely seizure‐free since surgery (ILAE 1a) (Figure 2).

During early postoperative months, a total of 10 patients were initially not seizure‐free. Of these, two (20%) patients experienced a running‐down phenomenon, in which seizures ultimately remitted at one‐year follow‐up. One patient (no. 16) experienced acute postoperative (APO) seizures, after which seizure freedom was maintained at one‐year postoperative follow‐up.

Two of six cases with ILAE 1a and one of the cases with ILAE 1 that experienced running‐down of seizures required antiepileptic drugs (AEDs) tapering at one‐year postoperative follow‐up (no. 2 tapered from 5 to 2 AEDs, no. 9 tapered from 3 to 2 AEDs, and no. 14 tapered from 4 to 3 AEDs) without seizure recurrence.

Of the eight patients who did not become seizure‐free at follow‐up, three (37.5%) patients underwent second epilepsy surgery including one (no. 6) with frontal resection following mapping of EZ by chronic subdural EEG recording and two (no. 5 and 7) with vagal nerve stimulator (VNS) implantation.

Five of 10 (50%) cases with seizure onset localization involving frontal lobe and three of six (50%) patients with seizure onset localization extending beyond the frontal lobe became seizure‐free (*P* = 1.00).

All of the four (100%) cases that harbored purely frontal MRI findings became seizure‐free (ILAE 1) after surgery. On the other hand, only two (33.3%) cases with neuroimaging evidence of a more extensive pathology and two (33.3%) cases that were MRI‐negative reported freedom from seizures at follow‐up (*P* = .07).

Three of six (50%) cases with histopathologic finding of FCD, three of five (60%) cases with no specific histopathologic changes, one (100%) case with dual pathology, one of two (50%) cases with no surgical specimen submitted, and none with glial scar became seizure‐free at 1 year postoperatively (*P* = .52).

Overall, there were no statistically significant differences in the baseline clinical, neuroimaging, surgical, and histopathologic variables in those who achieved seizure freedom at 1 postoperative year compared to those who did not.

## 4 DISCUSSION

Our study shows that frontal disconnection can result in seizure freedom, especially when the EZ is strictly limited to the ipsilateral frontal region, and the MRI shows an epileptogenic lesion that is purely frontal in location.

On reviewing the literature, there are two case reports^22^, ^23^ of frontal disconnection. One report described frontal disconnection performed on a 9‐year‐old boy with lesional DRE due to FCD with seizure freedom at 3‐year follow‐up. The other report was on frontal disconnection procedure done on a 17‐year‐old girl who previously failed to control her seizures after undergoing resection of frontal pilocytic astrocytoma. The frontal disconnection surgery has resulted in seizure freedom for follow‐up period of 16 months. In our surgical series, none of the patients had tumor; cases in which the presurgical MRI was suggestive of tumor were always offered resective surgery rather than disconnective surgery during our Epilepsy Management Conference.

Overall, our study has shown that half of patients who underwent frontal disconnection were seizure‐free at follow‐up. This is almost similar to those reported in studies involving longitudinal follow‐up of resective‐type frontal lobe surgery with resultant seizure freedom of 55.7%‐66% at 1 postoperative year.^4^, ^5^

Few studies though have evaluated the rate and stability of seizure outcome over time after frontal lobe resection.^4^, ^5^, ^6^, ^29^ Elsharkawy et al have reported seizure freedom of 54.6% at 6 postoperative months, 49.2% at 2 postoperative years, 47% at 5 postoperative years, and 41.9% at 10 postoperative years.^6^ Similarly, we speculate that seizure‐free state would remain stable with gradual slow decline in our series at longer postoperative follow‐up period.

Our series shows a lower seizure freedom rate in MRI‐negative patients, which is 33.3% at 1 postoperative year in comparison with that reported by Seo et al^8^ with a seizure freedom of 69% at 2 postoperative years in MRI‐negative frontal lobe epilepsy (FLE). However, there might be differences at baseline between our series and those in their study that needs to be clarified in another study comparing disconnection‐ to resection‐type surgery with a larger sample size before making inferences related to the surgical procedure.

Among patients who were not seizure‐free during first few postoperative months, we observed running‐down phenomena of seizures in 20% at 1‐year follow‐up.

Running‐down phenomena of seizures in FLE following frontal lobe surgery has been reported to occur in 9% at 2‐year follow‐up and 13.6% at 5‐year follow‐up.^6^

**FIGURE 3:**
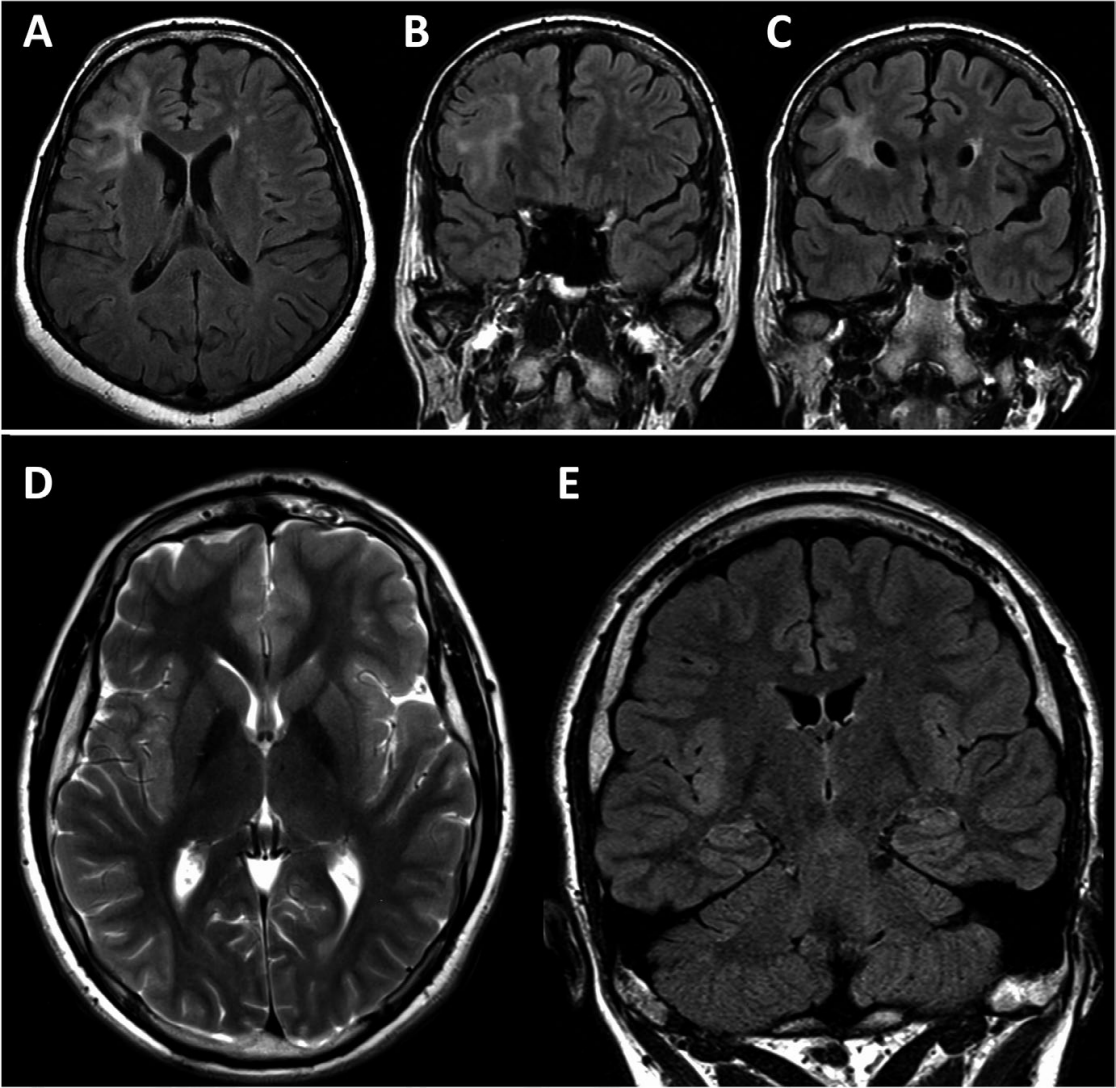
Presurgical MRI (A‐E) of two adult patients with focal cortical dysplasia type IIb. The first patient, a 38‐year‐old woman, had a large lesion involving the right frontal lobe as seen on axial and coronal planes of FLAIR (A‐C) images. In comparison, the lesion appears to be smaller and more localized to insular region on presurgical MRI including axial plane of T2‐weighted and coronal plane of FLAIR (D, E) images in the second patient, who is an 18‐year‐old boy. However, in this patient, invasive EEG revealed a wide epileptogenic zone involving the perirolandic area on dorsolateral and mesial surfaces

Of the FCD subtypes, FCD type IIb has been associated with good outcome as seizure freedom was achieved in 88% of patients undergoing focal resection in one study.^30^ However, three cases in our series had FCD type IIb with lesional MRI, where two patients (no. 1 and 2) had a large frontal lesion on MRI and the third patient (no. 3) had a lesion involving the insular region (Figure 3). Moreover, the second phase of evaluation with invasive EEG (performed in patients no. 1 and 3) has revealed a wide EZ surrounding the lesion that would need a large surgical resection (Table 1). So, disconnection surgery was offered in these cases as an alternative to a large resective surgery. In the third case, the EZ involved the perirolandic mesial and dorsolateral frontoparietal region and surgical decision was considered palliative as it involved isolated ipsilateral frontal disconnection without the adjacent eloquent areas in the perirolandic region. The first two patients with FCD type IIb had achieved seizure freedom at 1‐year follow‐up, while the third patient did not, which can be explained by our surgical approach that resulted in incomplete resection of the EZ; thus, not achieving much of a satisfactory palliative effect and need to be considered for reevaluation and possibly reoperation. Sarkis et al showed that seizure freedom outcome can be achieved even in patients with focal DRE due to FCD involving the rolandic region reaching up to 75% in patients with FCD type IIb compared to 44% in patients with other types of FCD suggesting that seizure outcome depends on the type of FCD lesion.^31^ They also showed that three patients with rolandic FCD type IIb and incomplete resection achieved seizure freedom following a second extensive surgical resection.^31^

A nonspecific pathological finding was a common diagnosis in biopsy obtained from edge of disconnected frontal lobe. Also, we noted that most of our MRI‐negative patients did not have a pathological diagnosis, in fact, none of them, except when one of the patients underwent a subsequent resective surgery revealing the diagnosis of FCD. Thus, it is likely that the pathological tissue was missed when biopsies were taken from edge of disconnected frontal lobe and we recommend obtaining more than one biopsy, including from edge of disconnected frontal lobe and another one from the mapped EZ in order to improve detection of a pathological finding that may have an implication in predicting seizure outcome in future studies.

Apart from neurological deficits resulting from involvement of eloquent areas in surgery, we could not find published reports on surgical‐related complications specifically in resection‐type frontal lobe surgeries. Thus, surgical‐related complications are underreported in literature. In this surgical series, we provided our data on surgical‐related complications; however, no inferences can be made about the rate of complication compared between resection‐ and disconnection‐type frontal lobe surgeries.

In conclusion, we showed that frontal disconnection surgery for DRE can result in seizure freedom in certain patients and that frontal lobe disconnection procedure is safe and has a limited complication rate.

## CONFLICTS OF INTEREST

None of the authors has any conflict of interest to disclose. We confirm that we have read the Journal's position on issues involved in ethical publication and affirm that this report is consistent with those guidelines.

## Supporting information

Supplementary Material
