# Supplementary material for: Frontal disconnection surgery for drug‐resistant epilepsy: Outcome in a series of 16 patients
Source: Epilepsia Open. 2020 Aug 14;5(3):475–86. doi: 10.1002/epi4.12424 (PMC7469852; doi:10.1002/epi4.12424)
Supplement: Supplementary file 1 — Supplementary Material [file EPI4-5-475-s001.docx]

**
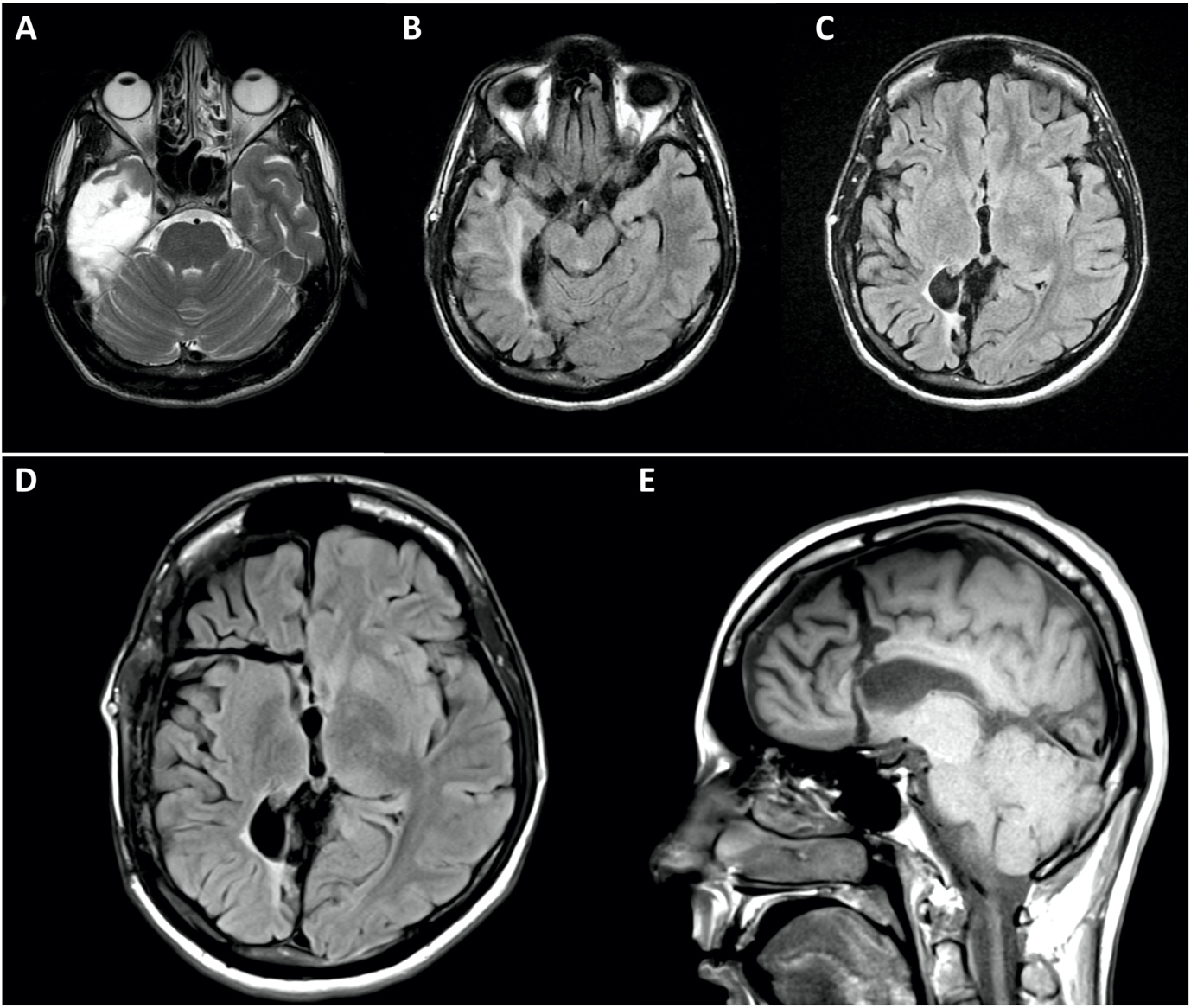
**

Figure S1: A 24-year-old gentleman who is status post right selective amygdalohippocampectomy continued to experience drug-resistant epilepsy arising from multiple foci in the right hemisphere, mostly in the right frontal lobe. Following detailed re-evaluation, he underwent right frontal disconnection combined with resection of residual right temporal tissue and multiple subpial transection at the right parietooccipital area. Histopathology revealed glial scar in both temporal and frontal specimens. Presurgical MRI including axial plane of T2-weighted (A) and fluid attenuated inversion recovery (FLAIR) images (B, C) revealed evidence of previous partial resection of right temporal lobe with encephalomalacia along the right temporal and occipital lobes and resultant ex-vacuo dilatation of the trigone and occipital horn. Postsurgical MRI including axial plane of FLAIR (D) and sagittal T1-weighted (E) images demonstrating the frontal disconnection line through the anterior corpus callosum.
